# Pyridine-2-carbaldehyde thio­semi­carbazone

**DOI:** 10.1107/S1600536809001962

**Published:** 2009-01-23

**Authors:** Li-Hua Song, Xiang Zhang, Kun Jiang, Sheng-Xiang Yang

**Affiliations:** a Huainan Union University, Huainan, Anhui 232038, People’s Republic of China; b Anhui Huainan Environmental Protection Agency, Huainan, Anhui 232038, People’s Republic of China

## Abstract

The asymmetric unit of the title compound, C_7_H_8_N_4_S, contains two independent mol­ecules with slightly different conformations; the dihedral angles between the pyridine ring and mean plane of the thio­semicarbazone unit in the two mol­ecules are 2.88 (5) and 6.30 (5)°. Inter­molecular N—H⋯N and N—H⋯S hydrogen bonds link the mol­ecules into layers parallel to the *ab* plane.

## Related literature

For the properties of thio­semicarbazones, see: Beraldo & Gambino (2004 ▶). For the crystal structure of a related compound, see: Gu *et al.* (2008 ▶).
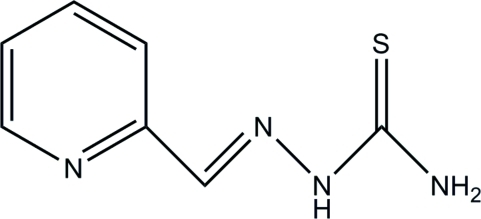


## Experimental

### 

#### Crystal data

C_7_H_8_N_4_S
*M* *_r_* = 180.23
Orthorhombic, 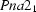

*a* = 20.725 (2) Å
*b* = 4.7857 (6) Å
*c* = 17.393 (2) Å
*V* = 1725.1 (4) Å^3^
*Z* = 8
Mo *K*α radiation
μ = 0.32 mm^−1^
*T* = 298 (2) K
0.45 × 0.20 × 0.19 mm

#### Data collection

Bruker SMART CCD area-detector diffractometer
Absorption correction: multi-scan (*SADABS*; Sheldrick, 1996 ▶) *T*_min_ = 0.868, *T*_max_ = 0.941
7296 measured reflections
2797 independent reflections
1951 reflections with *I* > 2σ(*I*)
*R*_int_ = 0.059

#### Refinement

*R*[*F* ^2^ > 2σ(*F* ^2^)] = 0.066
*wR*(*F* ^2^) = 0.165
*S* = 0.96
2797 reflections
218 parameters
1 restraint
H-atom parameters constrained
Δρ_max_ = 0.97 e Å^−3^
Δρ_min_ = −0.96 e Å^−3^
Absolute structure: Flack (1983 ▶), 1218 Friedel pairs
Flack parameter: 0.02 (19)

### 

Data collection: *SMART* (Siemens, 1996 ▶); cell refinement: *SAINT* (Siemens, 1996 ▶); data reduction: *SAINT*; program(s) used to solve structure: *SHELXS97* (Sheldrick, 2008 ▶); program(s) used to refine structure: *SHELXL97* (Sheldrick, 2008 ▶); molecular graphics: *SHELXTL* (Sheldrick, 2008 ▶); software used to prepare material for publication: *SHELXTL*.

## Supplementary Material

Crystal structure: contains datablocks I, global. DOI: 10.1107/S1600536809001962/cv2504sup1.cif

Structure factors: contains datablocks I. DOI: 10.1107/S1600536809001962/cv2504Isup2.hkl

Additional supplementary materials:  crystallographic information; 3D view; checkCIF report

## Figures and Tables

**Table 1:** Hydrogen-bond geometry (Å, °)

*D*—H⋯*A*	*D*—H	H⋯*A*	*D*⋯*A*	*D*—H⋯*A*
N1—H1⋯N8^i^	0.86	2.18	3.032 (8)	169
N3—H3*B*⋯S2^ii^	0.86	2.57	3.417 (6)	168
N7—H7*B*⋯S1^iii^	0.86	2.60	3.455 (7)	172
N5—H5⋯N4	0.86	2.36	3.199 (8)	164

Symmetry codes: (i) 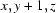; (ii) 
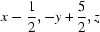
; (iii) 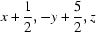.

**Fig. 1.**
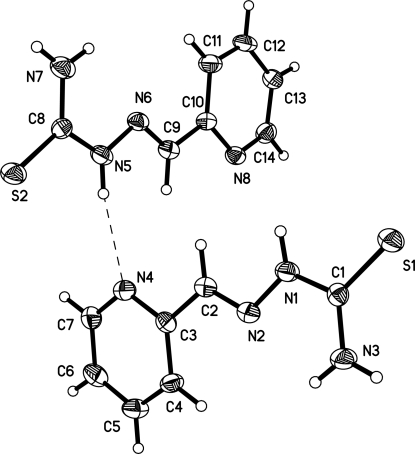
The content of asymmetric unit of the title compound showing the atomic numbering scheme and 30% probability displacement ellipsoids. Dashed line denotes hydrogen bond.

## supplementary crystallographic information

### Comment

Thiosemicarbazones have been known for many years to show a broad spectrum of therapeutic properties against a range of diseases, with antibacterial, antimalarial, antiviral and antitumour activities (Beraldo & Gambino, 2004), In this paper, we present the crystal structure of the title compound, (I).

In (I) (Fig. 1), the bond lengths and angles are normal and comparable to those observed in the reported compound (Gu *et al.*, 2008). The mean planes C10/C11/C12/C13/C14/N8 and C9/N6/N5 form a dihedral angle of 2.88 (5)°, while C3/C4/C5/C6/C7/N4 and C2/N2/N1 form a dihedral angle of 6.30 (5)°.

In the crystal, the intermolecular N—H···N and N—H···S hydrogen bonds (Table 1) link the molecules into layers parallel to *ab* plane.

### Experimental

Pyridine-2-carbaldehyde (0.5 mmol), thiosemicarbazide (0.5 mmol) and 20 ml ethanol were mixed in 50 ml flask. After stirring 30 min at 373 K, the resulting mixture was recrystalized from ethanol, affording the title compound as a orange crystalline solid. Elemental analysis: calculated for C_7_H_8_N_4_S: C 46.65, H 4.47, N 31.09%; found: C46.58, H 4.56, N 31.11%.

### Refinement

All H atoms were placed in geometrically idealized positions (N—H 0.86 Å, C—H 0.93 Å) and treated as riding on their parent atoms, with *U*_iso_(H) = 1.2 *U*_eq_(C,*N*).

### Crystal data

**Table:**

C_7_H_8_N_4_S	*D*_x_ = 1.388 Mg m^−^^3^
*M**_r_* = 180.23	Mo *K*α radiation, λ = 0.71073 Å
Orthorhombic, *P**n**a*2_1_	Cell parameters from 1607 reflections
*a* = 20.725 (2) Å	θ = 2.3–21.6°
*b* = 4.7857 (6) Å	µ = 0.32 mm^−^^1^
*c* = 17.393 (2) Å	*T* = 298 K
*V* = 1725.1 (4) Å^3^	Block, orange
*Z* = 8	0.45 × 0.20 × 0.19 mm
*F*(000) = 752	

### Data collection

**Table:**

Bruker SMART CCD area-detector diffractometer	2797 independent reflections
Radiation source: fine-focus sealed tube	1951 reflections with *I* > 2σ(*I*)
graphite	*R*_int_ = 0.059
φ and ω scans	θ_max_ = 25.0°, θ_min_ = 2.3°
Absorption correction: multi-scan (*SADABS*; Sheldrick, 1996)	*h* = −24→20
*T*_min_ = 0.868, *T*_max_ = 0.941	*k* = −5→5
7296 measured reflections	*l* = −20→17

### Refinement

**Table:**

Refinement on *F*^2^	Secondary atom site location: difference Fourier map
Least-squares matrix: full	Hydrogen site location: inferred from neighbouring sites
*R*[*F*^2^ > 2σ(*F*^2^)] = 0.066	H-atom parameters constrained
*wR*(*F*^2^) = 0.165	*w* = 1/[σ^2^(*F*_o_^2^) + (0.0511*P*)^2^ + 6.2026*P*] where *P* = (*F*_o_^2^ + 2*F*_c_^2^)/3
*S* = 0.96	(Δ/σ)_max_ = 0.001
2797 reflections	Δρ_max_ = 0.97 e Å^−^^3^
218 parameters	Δρ_min_ = −0.96 e Å^−^^3^
1 restraint	Absolute structure: Flack (1983), 1218 Friedel pairs
Primary atom site location: structure-invariant direct methods	Flack parameter: 0.02 (19)

### Special details

**Table:**

Geometry. All e.s.d.'s (except the e.s.d. in the dihedral angle between two l.s. planes) are estimated using the full covariance matrix. The cell e.s.d.'s are taken into account individually in the estimation of e.s.d.'s in distances, angles and torsion angles; correlations between e.s.d.'s in cell parameters are only used when they are defined by crystal symmetry. An approximate (isotropic) treatment of cell e.s.d.'s is used for estimating e.s.d.'s involving l.s. planes.
Refinement. Refinement of *F*^2^ against ALL reflections. The weighted *R*-factor *wR* and goodness of fit *S* are based on *F*^2^, conventional *R*-factors *R* are based on *F*, with *F* set to zero for negative *F*^2^. The threshold expression of *F*^2^ > σ(*F*^2^) is used only for calculating *R*-factors(gt) *etc*. and is not relevant to the choice of reflections for refinement. *R*-factors based on *F*^2^ are statistically about twice as large as those based on *F*, and *R*- factors based on ALL data will be even larger.

### Fractional atomic coordinates and isotropic or equivalent isotropic displacement parameters (Å^2^)

**Table:**

	*x*	*y*	*z*	*U*_iso_*/*U*_eq_	
N8	0.6201 (3)	−0.1518 (12)	0.5432 (3)	0.0387 (14)	
N1	0.5188 (3)	0.9637 (13)	0.4216 (3)	0.0405 (15)	
H1	0.5464	0.9533	0.4584	0.049*	
N2	0.5264 (3)	0.8019 (12)	0.3566 (3)	0.0385 (14)	
N3	0.4295 (3)	1.1564 (14)	0.3672 (3)	0.0514 (18)	
H3A	0.4376	1.0591	0.3267	0.062*	
H3B	0.3965	1.2651	0.3682	0.062*	
N4	0.6451 (3)	0.3132 (13)	0.2989 (3)	0.0443 (15)	
N5	0.7451 (3)	0.5151 (13)	0.4275 (3)	0.0411 (14)	
H5	0.7213	0.4881	0.3877	0.049*	
N6	0.7334 (3)	0.3664 (12)	0.4937 (3)	0.0371 (14)	
N7	0.8267 (3)	0.7425 (15)	0.4879 (4)	0.0493 (17)	
H7A	0.8170	0.6506	0.5288	0.059*	
H7B	0.8580	0.8603	0.4885	0.059*	
S1	0.45768 (9)	1.3182 (4)	0.50951 (10)	0.0483 (5)	
S2	0.80649 (9)	0.8767 (4)	0.34251 (11)	0.0480 (5)	
C1	0.4677 (3)	1.1390 (15)	0.4274 (4)	0.0352 (16)	
C2	0.5761 (3)	0.6438 (14)	0.3561 (4)	0.0383 (17)	
H2	0.6046	0.6495	0.3975	0.046*	
C3	0.5892 (3)	0.4557 (14)	0.2926 (4)	0.0362 (16)	
C4	0.5478 (3)	0.4143 (16)	0.2310 (4)	0.0438 (18)	
H4	0.5097	0.5157	0.2272	0.053*	
C5	0.5637 (4)	0.2228 (17)	0.1758 (5)	0.054 (2)	
H5A	0.5367	0.1944	0.1338	0.064*	
C6	0.6199 (4)	0.0727 (17)	0.1829 (5)	0.054 (2)	
H6	0.6310	−0.0623	0.1468	0.065*	
C7	0.6591 (4)	0.1270 (17)	0.2447 (4)	0.049 (2)	
H7	0.6976	0.0283	0.2488	0.058*	
C8	0.7935 (3)	0.7027 (15)	0.4244 (4)	0.0365 (17)	
C9	0.6886 (3)	0.1868 (15)	0.4895 (4)	0.0383 (18)	
H9	0.6667	0.1636	0.4432	0.046*	
C10	0.6706 (3)	0.0160 (14)	0.5554 (4)	0.0351 (16)	
C11	0.7028 (4)	0.0259 (17)	0.6248 (4)	0.049 (2)	
H11	0.7375	0.1461	0.6317	0.058*	
C12	0.6825 (4)	−0.1454 (17)	0.6836 (5)	0.053 (2)	
H12	0.7030	−0.1408	0.7311	0.063*	
C13	0.6313 (3)	−0.3245 (16)	0.6709 (4)	0.0471 (19)	
H13	0.6166	−0.4438	0.7093	0.057*	
C14	0.6029 (3)	−0.3205 (17)	0.6003 (5)	0.047 (2)	
H14	0.5691	−0.4445	0.5914	0.057*	

### Atomic displacement parameters (Å^2^)

**Table:**

	*U*^11^	*U*^22^	*U*^33^	*U*^12^	*U*^13^	*U*^23^
N8	0.044 (3)	0.040 (3)	0.033 (3)	0.004 (3)	−0.004 (3)	0.003 (3)
N1	0.044 (3)	0.044 (4)	0.033 (3)	0.003 (3)	−0.006 (3)	−0.004 (3)
N2	0.043 (3)	0.039 (3)	0.034 (4)	−0.002 (3)	−0.003 (3)	−0.001 (3)
N3	0.049 (4)	0.064 (4)	0.041 (4)	0.013 (3)	−0.013 (3)	−0.018 (3)
N4	0.043 (3)	0.050 (4)	0.040 (4)	0.002 (3)	−0.005 (3)	−0.002 (3)
N5	0.048 (4)	0.047 (4)	0.029 (3)	0.001 (3)	−0.002 (3)	−0.003 (3)
N6	0.042 (3)	0.037 (3)	0.032 (4)	−0.001 (3)	−0.003 (2)	−0.007 (3)
N7	0.053 (3)	0.057 (4)	0.038 (4)	0.014 (3)	−0.003 (3)	−0.013 (3)
S1	0.0529 (10)	0.0535 (11)	0.0384 (11)	0.0042 (10)	−0.0040 (9)	−0.0095 (10)
S2	0.0506 (10)	0.0558 (11)	0.0377 (10)	0.0049 (10)	−0.0046 (9)	−0.0140 (10)
C1	0.035 (4)	0.034 (4)	0.037 (4)	−0.006 (3)	0.003 (3)	0.001 (3)
C2	0.040 (4)	0.040 (4)	0.034 (4)	0.000 (3)	−0.003 (3)	0.001 (3)
C3	0.043 (4)	0.033 (4)	0.033 (4)	0.001 (3)	0.003 (3)	−0.001 (3)
C4	0.040 (4)	0.050 (5)	0.041 (4)	0.010 (4)	−0.010 (3)	−0.003 (4)
C5	0.058 (5)	0.061 (5)	0.042 (5)	0.000 (5)	−0.014 (4)	−0.004 (4)
C6	0.070 (5)	0.056 (5)	0.038 (5)	0.003 (4)	0.002 (4)	−0.008 (4)
C7	0.044 (4)	0.058 (5)	0.044 (5)	0.008 (4)	0.005 (4)	−0.002 (4)
C8	0.034 (4)	0.037 (4)	0.038 (4)	−0.010 (3)	0.000 (3)	−0.004 (3)
C9	0.043 (4)	0.039 (4)	0.033 (4)	0.001 (3)	−0.003 (3)	−0.002 (3)
C10	0.039 (4)	0.034 (4)	0.033 (4)	0.000 (3)	−0.005 (3)	0.001 (3)
C11	0.050 (4)	0.056 (5)	0.040 (5)	0.008 (4)	−0.011 (3)	−0.006 (4)
C12	0.060 (5)	0.067 (6)	0.031 (4)	−0.006 (4)	−0.011 (4)	−0.006 (4)
C13	0.048 (4)	0.052 (5)	0.042 (5)	0.001 (4)	0.000 (3)	−0.013 (4)
C14	0.041 (4)	0.050 (5)	0.050 (5)	0.003 (4)	−0.002 (3)	0.003 (4)

### Geometric parameters (Å, °)

**Table:**

N8—C14	1.328 (9)	C2—C3	1.450 (9)
N8—C10	1.336 (8)	C2—H2	0.9300
N1—C1	1.354 (8)	C3—C4	1.388 (9)
N1—N2	1.380 (8)	C4—C5	1.367 (10)
N1—H1	0.8600	C4—H4	0.9300
N2—C2	1.278 (8)	C5—C6	1.373 (10)
N3—C1	1.316 (9)	C5—H5A	0.9300
N3—H3A	0.8600	C6—C7	1.372 (10)
N3—H3B	0.8600	C6—H6	0.9300
N4—C7	1.330 (9)	C7—H7	0.9300
N4—C3	1.348 (8)	C9—C10	1.458 (10)
N5—C8	1.347 (8)	C9—H9	0.9300
N5—N6	1.375 (8)	C10—C11	1.379 (9)
N5—H5	0.8600	C11—C12	1.376 (11)
N6—C9	1.267 (9)	C11—H11	0.9300
N7—C8	1.314 (9)	C12—C13	1.382 (10)
N7—H7A	0.8600	C12—H12	0.9300
N7—H7B	0.8600	C13—C14	1.362 (10)
S1—C1	1.679 (7)	C13—H13	0.9300
S2—C8	1.671 (8)	C14—H14	0.9300
			
C14—N8—C10	117.2 (6)	C4—C5—H5A	120.3
C1—N1—N2	119.9 (6)	C6—C5—H5A	120.3
C1—N1—H1	120.1	C7—C6—C5	118.3 (7)
N2—N1—H1	120.1	C7—C6—H6	120.8
C2—N2—N1	115.5 (6)	C5—C6—H6	120.8
C1—N3—H3A	120.0	N4—C7—C6	123.5 (7)
C1—N3—H3B	120.0	N4—C7—H7	118.2
H3A—N3—H3B	120.0	C6—C7—H7	118.2
C7—N4—C3	118.0 (6)	N7—C8—N5	116.9 (7)
C8—N5—N6	120.7 (6)	N7—C8—S2	124.0 (6)
C8—N5—H5	119.7	N5—C8—S2	119.1 (5)
N6—N5—H5	119.7	N6—C9—C10	121.4 (7)
C9—N6—N5	115.6 (6)	N6—C9—H9	119.3
C8—N7—H7A	120.0	C10—C9—H9	119.3
C8—N7—H7B	120.0	N8—C10—C11	122.6 (7)
H7A—N7—H7B	120.0	N8—C10—C9	114.3 (6)
N3—C1—N1	116.8 (6)	C11—C10—C9	123.1 (7)
N3—C1—S1	124.8 (6)	C12—C11—C10	118.8 (7)
N1—C1—S1	118.5 (5)	C12—C11—H11	120.6
N2—C2—C3	121.5 (6)	C10—C11—H11	120.6
N2—C2—H2	119.2	C11—C12—C13	119.0 (7)
C3—C2—H2	119.2	C11—C12—H12	120.5
N4—C3—C4	121.4 (6)	C13—C12—H12	120.5
N4—C3—C2	114.4 (6)	C14—C13—C12	117.8 (8)
C4—C3—C2	124.2 (6)	C14—C13—H13	121.1
C5—C4—C3	119.3 (7)	C12—C13—H13	121.1
C5—C4—H4	120.4	N8—C14—C13	124.5 (8)
C3—C4—H4	120.4	N8—C14—H14	117.8
C4—C5—C6	119.5 (7)	C13—C14—H14	117.8

### Hydrogen-bond geometry (Å, °)

**Table:**

*D*—H···*A*	*D*—H	H···*A*	*D*···*A*	*D*—H···*A*
N1—H1···N8^i^	0.86	2.18	3.032 (8)	169
N3—H3B···S2^ii^	0.86	2.57	3.417 (6)	168
N7—H7B···S1^iii^	0.86	2.60	3.455 (7)	172
N5—H5···N4	0.86	2.36	3.199 (8)	164

Symmetry codes: (i) *x*, *y*+1, *z*; (ii) *x*−1/2, −*y*+5/2, *z*; (iii) *x*+1/2, −*y*+5/2, *z*.
